# Molecular Cloning and Characterization of Novel *Morus alba* Germin-Like Protein Gene Which Encodes for a Silkworm Gut Digestion-Resistant Antimicrobial Protein

**DOI:** 10.1371/journal.pone.0050900

**Published:** 2012-12-19

**Authors:** Bharat Bhusan Patnaik, Dong Hyun Kim, Seung Han Oh, Yong-Su Song, Nguyen Dang Minh Chanh, Jong Sun Kim, Woo-jin Jung, Atul Kumar Saha, Bharat Bhushan Bindroo, Yeon Soo Han

**Affiliations:** 1 Division of Plant Biotechnology, College of Agriculture and Life Sciences, Chonnam National University, Gwangju, South Korea; 2 Division of Applied Bioscience and Biotechnology, Institute of Environmentally-Friendly Agriculture, Chonnam National University, Gwangju, Republic of Korea; 3 Institute of Insect and Sericultural Research, Jeonnam Agricultural Research and Extension Service, Jangseong, Republic of Korea; 4 Central Sericultural Research and Training Institute, Central Silk Board (Govt. of India), Berhampore, West Bengal, India; University of South Florida College of Medicine, United States of America

## Abstract

**Background:**

Silkworm fecal matter is considered one of the richest sources of antimicrobial and antiviral protein (substances) and such economically feasible and eco-friendly proteins acting as secondary metabolites from the insect system can be explored for their practical utility in conferring broad spectrum disease resistance against pathogenic microbial specimens.

**Methodology/Principal Findings:**

Silkworm fecal matter extracts prepared in 0.02 M phosphate buffer saline (pH 7.4), at a temperature of 60°C was subjected to 40% saturated ammonium sulphate precipitation and purified by gel-filtration chromatography (GFC). SDS-PAGE under denaturing conditions showed a single band at about 21.5 kDa. The peak fraction, thus obtained by GFC wastested for homogeneityusing C18reverse-phase high performance liquid chromatography (HPLC). The activity of the purified protein was tested against selected Gram +/− bacteria and phytopathogenic *Fusarium* species with concentration-dependent inhibitionrelationship. The purified bioactive protein was subjected to matrix-assisted laser desorption and ionization-time of flight mass spectrometry (MALDI-TOF-MS) and N-terminal sequencing by Edman degradation towards its identification. The N-terminal first 18 amino acid sequence following the predicted signal peptide showed homology to plant germin-like proteins (Glp). In order to characterize the full-length gene sequence in detail, the partial cDNA was cloned and sequenced using degenerate primers, followed by 5′- and 3′-rapid amplification of cDNA ends (RACE-PCR). The full-length cDNA sequence composed of 630 bp encoding 209 amino acids and corresponded to germin-like proteins (Glps) involved in plant development and defense.

**Conclusions/Significance:**

The study reports, characterization of novel Glpbelonging to subfamily 3 from *M. alba* by the purification of mature active protein from silkworm fecal matter. The N-terminal amino acid sequence of the purified protein was found similar to the deduced amino acid sequence (without the transit peptide sequence) of the full length cDNA from *M. alba*.

## Introduction

Sericulture is one of the oldest agro-based industries in the world. From time to time, sericulture practices have undergone changes to improve productivity. Though Indian silk industry has created an impact in global silk scenario, emerging as the second largest producer, it has to withstand major constraints in the form of disease development and mortality. The loss due to silkworm diseases in India is reported at 30–40%, a significant proportion of which is attributed to baculoviruses, especially *Bombyx mori*nucleopolyhedrosis virus (BmNPV) [1]and minor forms of bacterial flacherie, septicemia and toxemia caused by bacteria as *Bacillus*, *Staphylococcus*, *Streptococcus*, *Proteus* etc [2], [3].

Researchers have characterized antiviral proteins from midgut and haemolymph of silkworm, *Bombyx mori*.The inhibitory and antiviral properties of digestive juice of *Bombyx mori* larvae have been investigated and an unknown substance of high molecular weight in the gut juice has been reported that could inactivate nuclear polyhedrosis virus *in vitro* [4], [5]. A red fluorescent protein (RFP) from the digestive juice of *B. mori* has been reported and was found to inactivate the NPV of *B. mori*, *in vitro* [6], [7]. Others have reported the agglutination reaction of NPV and RFP [8], [9]. RFP as a wholehas been suggested as having more than one midgut protein and the antiviral activity is attributable to Phospholipase-C (PLC) [10]. The role of Bmlipase-1 (*Bombyx mori* Lipase-1) and BmSP-2 (*Bombyx mori* Serine Protease-2) from digestive juice having strong antiviral activity against BmNPV has also been demonstrated [1], [11]. Such substances with some pattern recognition proteins in haemolymph have presented unity and diversity in antiviral substance mechanisms.

Silkworm, *Bombyx mori* L. fecal matter has been identified as having beneficial effects against different pathophysiological states[12], [13]. Antimicrobial peptide discovery from feces of normal healthy silkworms provides with a logistical option towards invigorating drugs of biological origin that shows “drug-likeness and biological friendliness” in comparison to synthetic drug candidates and would be eco-friendly and aqua-soluble, preventing the residual anxiety and toxicity. This would have significant impact as pathogenic microbes have gained resistance to various innovative drugs such as Amoxicillin and Ciprofloxacin [14], [15]. Additionally, the drug toxicity to the host tissue remains a contentious issue with its obvious cost at the market, which leads to inequality in the distribution and procurement. This would guarantee a surplus for the sericulture farms as the drug candidates would be obtained as a by-product of seri-farming practices. It could also be envisioned that the drug candidate can be used as an effective antimicrobial agent in silk insect producing farms.

So far, literature is sparse in relation to antiviral substances isolated and purified from silkworm excrement. Antiviral substances were observed to inactivate many types of cells in suspension and recognized mannose on cell surface leading to aggregation. Antiviral proteins have been purified from silkworm fecal matter and reported to be glycoproteins [16]. The proteins purified have been reported to act as an antiviral against enveloped viruses such as human immunodeficiency virus (HIV), Sendai virus (HVJ), herpes simplex virus [17] and lately NPV [13] and also antibacterial against infectious clinical strains such as *Staphylococcus aureus*, *Bacillus subtilis*, *Streptococcus hemolyticus*, *Salmonella typhi*, *E. coli*, *Pseudomonas aeruginosa and Klebsiella Pneumoniae* [18]. Antiviral activity of L4-1 against HVJ-LLC-MK2 cell system depends on light irradiation and is inhibited by sodium sulphite and anaerobic conditions. The activity was due to damage to viral proteins caused by reactive oxygen species produced by L4-1 [19], [20].

An enveloped animal virus, vesicular stomatitis virus (VSV, an RNA virus), was also inhibited by chlorophyll derivatives (CpD) from silkworm excreta in photodynamic antimicrobial chemotherapy (PACT) [21]. Further, inhibition of viral RNA synthesis was observed in host cells inoculated with CpD-PACT treated virus. In addition, the CpD acts as an efficient photodynamic antiviral agent. CpD was also studied towards evaluation of its inhibitory effects on a mouse retrovirus isolated from the gross leukemia virus (GLV)-producing TGV cell line and was associated with immediate inhibition of GLV associated reverse transcriptase (RT) activity, suggesting its potential as an anti-retroviral agent. [19]. In addition, the methanolic extracts of silkworm fecal matter showed cytotoxic effects on HT-29 human colon cancer cells by induction of apoptosis involving mitochondrial-mediated pathway [22].

Bioactive principles of silkworm feces have been explored in the field of medicine against various traditional infections in China [23]. Silkworm midgut membrane protein (P252) that binds chlorophyllide forming a red fluorescent protein (RFP) has been shown to have significant antimicrobial activity [24]. Silkworm excretory-RFP associated with two photochromic moieties as tetrapyrrole –I (TP-I) and tetrapyrrole –II (TP-II) and having broad-spectrum antimicrobial functions against some common bacteria and fungus have been also reported [25], [26]. The oral administration of RFPs has proved beneficial as it led to significant decrease in the incidence of nucleopolyhedrosis in silkworms [27].

Researchers in the thrust area have suggested immediate attention towards interdisciplinary investigations to examine the silkworm fecal matter for the presence of novel bioactive proteins. Complete characterization of antiviral and antimicrobial fractions will hopefully provide the sericulture industry a realized means to reap benefits through minimizing crop loss and would provide scope and immediate attention to screen different groups of pathogenic microbes, thus providing pharmacological significance. The strategy of characterizing the gene from the fecal extract having functional relevance in the insect or the food plant and eventually impacting plant-insect relations, seems a novel one and elucidation of Glp class of genes belonging to *M. alba* is substantive of the rich benefits of exploring the natural resource.

## Materials and Methods

### Collection of source materials

The 5^th^ instar 3^rd^ day larvae were used for collection of fecal matter. The fecal matter was procured from Silk Insects Research Centre, Jeonnamdo and was air-dried at room temperature and the leaves, dust and other contaminants were manually separated. The above cleaned silkworm fecal matter was stored at −20°C in a closed polythene container until they were used for further analysis.

### Chemicals

All chemicals used for the experiments were of analytical grade, obtained from Sigma Chemical Co. (St. Louis, MO, USA) until otherwise mentioned in the text.

### Bacterial and fungal strains

The Gram positive strains, viz., *Bacillus cereus* B33 (National centre for biotechnology information (NCBI) accession number FJ483513), *Bacillus subtilis* EG1 (NCBI accession number FJ483514) and Gram (-) bacterial strains as *Serratia marcescens* PRC-5 (NCBI accession number JN816402), *Salmonella enterica* Korea agricultural culture collection (KACC) 10763 (serovar enteridis), *Pseudomonas rhodesiae* NO5 (NCBI accession number FJ462694), *Pseudomonas entomophila* MG23S and *Aeromonas hydrophila* AKR 1 (NCBI accession number FJ462702) were used towards evaluation of the antibacterial activity of the purified protein. The fungal strains used for the assessment of antifungal status of the purified protein were *Fusarium oxysporum* KACC 40032 and *Fusarium solani* KACC 40384. These strains were maintained at Division of Applied Bioscience and Biotechnology, Chonnam National University, Gwangju, South Korea and the in-house facility was used towards the study.

Standard antibiotics as Neomycin and Chloramphenicol were used as antibacterial and Cycloheximide (M/s Sigma Chemicals Co., St. Louis, MO, USA) as antifungal agent acting as positive control in the assay design.

### Isolation and partial purification

The isolation and partial purification of bioactive protein(s) from silkworm fecal matter was conducted as described in [13] with slight modifications. Silkworm fecal matter (60 g) was powdered using a mortar. It was mixed with 0.02 M phosphate buffer saline (PBS), pH 7.4 and was continuously stirred for ∼40 hrs at 60°C. The preparation was filtered using Whatman filter paper no. 1 (11.0 cm diameter), and the filtrate was centrifuged for 20 min at 4000 rpm using refrigerated centrifuge at 4°C. The supernatant was used for further purification by subjecting it to 40% saturated ammonium sulphate precipitation. The preparation was incubated in ice for 90 min. Then, it was centrifuged at 10,000 rpm for 15 min at 4°C. The precipitate obtained from the above preparation was dissolved in an appropriate volume of 0.02 M phosphate buffer saline, pH 7.4 and was dialyzed against the same buffer using a dialysis membrane having pore size of 110, average flat width 33 mm, average diameter 21 mm when full and flow rate 3.63 ml/cm (Sigma Chemical Co., St. Louis, MO, USA) for a duration of 48 hrs. The dialysate was subjected to lyophilization and lyophilized protein was applied to column chromatography using silica gel ‘G’ as a matrix (mesh number 60/120; column 0.5×40 cm, obtained from M/s Acme's Laboratory Chemicals).

The column eluates were further purified by performing gel filtration chromatography (GFC) using Sephadex G-75 having a fractionation range of 3×10^3^–7×10^4^ as matrix (GE Healthcare, Uppsala, Sweden) at 4°C. Separation was accomplished by passing the eluates through C column (1.60×70 cm) at a flow rate of 0.1 ml/min and 500 µl sample volume. The gel-filtration chromatography setup was run on an Acta design fast performance liquid chromatography (FPLC) attached to Unicorn 5.0 software (Amersham Biosciences, Uppsala, Sweden). Protein peaks were detected by monitoring absorbance at 280 nm on UV-Vis flow cell of the system. Purified protein peak fractions (2 ml) were collected, pooled, lyophilized and stored at 4°C.

### High-performance liquid chromatography (HPLC) analysis of purified protein

The purified active fraction from GFC was subjected to analytical HPLC (Shimadzu LC-10 AD, Shimadzu Scientific Instruments, Japan) to confirm the purity. The purification was accomplished in a reverse-phase C18 column (Varian Pursuit XRs5, Agilent Technologies, Santa Clara, CA) of 250×4.6 mm and 5 µ particle size with a linear gradient of 30–70% acetonitrile/0.1% tri-fluoroacetic acid (TFA) in water at a flow rate of 0.8 ml/min. The sample injection volume was 25 µl with column oven temperature at 25°C. The protein peaks were detected at a wavelength of 280 nm in a diode array detector. Preparative HPLC purification was conducted in a reverse-phase C18 column (Varian Pursuit XRs, Agilent Technologies, Santa Clara, CA) of 250×10 mm and 5 µ particle size with a linear gradient of 30–70% acetonitrile/0.1% TFA in water at a flow rate of 4 ml/min. The sample injection volume was 200 µl with column oven temperature at 25°C. The protein peaks were detected at a wavelength of 280 nm in a diode array detector.

### Protein estimation

Protein content of partially purified and purified bioactive fractions was estimated [28]. In addition, the absorbance assay A_280_ method was used to estimate the protein concentration in the collected peak fractions.

### Electrophoresis under denaturing and native conditions

Sodium dodecyl sulfate polyacrylamide gel electrophoresis (SDS-PAGE) was carried out by using 12% resolving and 5% stacking gel [29] at a consistent running voltage of 100 V for 95 min. The protein bands in the gel were visualized by staining with Coomassie brilliant blue and destained with solution containing 5% methanol and 7% acetic acid. For silver staining, the protein was fixed in 50% methanol, 10% acetic acid in distilled water (DW) for 30 min. followed in 5% methanol, 7% acetic acid in DW for 30 min. The fixation step was followed with overnight wash in large volume of DW and staining in dithiothreitol (DTT) in DW for 30 min. and silver nitrate (0.1%) in DW for 10 min. The protein bands were developed by adding 50 µl, 37% formaldehyde in 100 ml of 3% sodium carbonate for 5–10 min or until brown color develops.

Gel electrophoresis under non-denaturing conditions (Native-PAGE) was carried out by using 12% resolving and 5% stacking gel at a running voltage of 100 V for 95 min in a cooling system. The sample buffer contained 5.55 ml DW, 1.25 ml Tris-HCl (0.5 M), pH 6.8, 3.0 ml glycerol and 0.2 ml of bromophenol blue (0.5%) as per the instructions (BioRad Laboratories, Hercules, CA). Coomassie blue staining and silver staining for visualization of the proteins in the gel was as discussed before in this section.

### Evaluation of antibacterial activity and minimum inhibitory concentration (MIC)

The antibacterial activity of novel protein purified from silkworm fecal extract was carried out by agar well diffusion method. For the assay, 25 ml of sterile nutrient agar medium (BD Difco™ ) was poured into sterile culture plates and allowed to solidify. The plates were incubated at 37°C for 24 hr to check for sterility. The bacterial cultures (200 µl) were spread separately on the agar medium. The wells (8 mm diameter) were made using stainless steel sterilized cork borer under aseptic conditions. The purified protein at a concentration of 2.5, 5, 10, 25 and 50 µg in phosphate buffer (pH 7.4) was loaded into the corresponding wells. Neomycin and Chloramphenicol at a concentration of 25 µg/100 µl was loaded as positive control and 0.02 M PBS, pH 7.4 acted as negative control. The plates were kept at 4°C for diffusion of the protein and then transferred to incubator at 37°C for 24 hr. The zone of complete inhibition of bacteria (diameter) was measured around each well. The results were expressed as mean ± SE of three replicates in each test. The MIC of purified bioactive protein was determined by micro-dilution method in nutrient broth [30].

### Antifungal activity assay

The antifungal activity of the novel protein was assessed quantitatively by studying the fungal growth inhibition using the microtiter plate assay [31]. The fungal spore suspension (0.1 ml, grown for 3 days in 10 ml of nutrient dextrose agar) was mixed with half strength potato-dextrose broth containing 10^4^ spores/ml. Wells of microtiter plates filled with different concentrations of protein (5, 10, 25, 50 and 60 µg/µl) were mixed with the spore suspension and were studied for the inhibition of spore germination at 0 hr, 1, 2, 3 and 4 day intervals by measuring absorbance at 620 nm. The percentage of inhibition (IR%) was calculated based on percentage inhibition of radial growth (PIRG%) as follows:
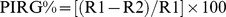
Where, R1 = radial growth in control

R2 = radial growth in treatment

The present experiment was also used to determine the inhibitory concentration (IC_50_) showing protein concentration required for 50% growth inhibition by probit analysis [32], [33]. Experiments were performed at least in three replicates.

### Statistical analysis

All experimental treatments were subjected to one-way ANOVA and Tukey's multiple range tests and are valued at 95% confidence (P<0.05) level. The statistical analysis was performed using the SAS (Statistical Analysis Software) package 9.1.3.

### Effect of pH and temperature on the activity of purified protein

The purified protein was incubated with an equal volume of 0.02 M buffers of desired pH (2 to 12) for 6 hs at 4°C. The 50 µl of incubated protein was used to determine the anti-bacterial zone of inhibition against *B. subtilis* strain according to the standard assay procedure as described earlier. A control was maintained to compare the effect of pH on the protein. The inhibition rate (%) was calculated as previously described.

To study the temperature-dependent activity, the purified protein (50 µg) in 0.02 M sodium phosphate buffer, pH 7.4 was taken in different eppendorf tubes and incubated at desired temperatures in a thermostat water bath for about 30 min. Then the incubated protein solutions were cooled to room temperature and were used to determine the anti-bacterial zone of inhibition against B. subtilis strain as described earlier. The inhibition rate (%) was calculated as previously described.

### Determination of intact molecular mass and protein identification

The HPLC purified protein samples was used for intact mass determination using matrix-assisted laser desorption and ionization-time of flight mass spectrometer (MALDI-MS). Sample concentrated by speed-vac was diluted with water and mixed with matrix solution (Sinapinic acid) at a ratio of 1∶1 (v/v). A one microliter portion was spotted onto the stainless steel target plate and dried in air. The MALDI-TOF-MS analysis was performed using a voyager-DE-STR MALDI-TOF mass spectrometer (Applied Biosystems, Farmington, MA, USA). The instrument was operated in linear mode of 25 kV accelerating voltage and 400 ns ion extraction delay with the nitrogen laser working at 337 nm and 3 Hz. Thousand shots/sec of laser of 35% strength were accumulated per spectrum. Internal calibration was firstly performed on the samples premixed with human rEPO using its doubly and singly charged peaks (m/z 14508.73, 28756.33) as well as its dimer and trimer peaks (57505.26, 84746.84 m/z). In this way, four strong and sharp peaks were assigned to human rEPO.

For protein identification by in-gel digestion MALDI MS, the purified protein was separated by SDS-PAGE (Bio-Rad Mini-PROTEAN II; 70×100×0.7 mm gels) as recommended by the manufacturer and stained with Coomassie Brilliant Blue R-250. The protein band was excised, transferred and deposited directly onto the MALDI probe. The MALDI probe was rehydrated by addition of a small volume of digestion buffer (12.5 ng/µl of trypsin in 50 mM NH_4_HCO_3_). The digestion was accomplished in a closed humid chamber and incubated at 30°C for 20 hrs. Delayed-extraction MALDI mass spectra were recorded on a REFLEX reflectron time-of-flight mass spectrometer (Voyager DE-STR, Applied Biosystems, Farmington, MA, USA). Internal mass calibration of peptide mass maps was done using bovine serum albumin (BSA). Raw data was refined by software data explorer, including baseline correction, noise filter and peak de-isotoping.

Refined peaks from data explorer were submitted to online server Mascot (http://www.matrixscience.com) and Protein Prospector (http://prospector.ucsf.edu) server. Search parameters were as follows: non-redundant database (NCBInr); all taxa; 0–300 kDa; pI 0–14; one missed cleavage site; cysteine residues modified with iodoacetamide; serine and threonine phosphorylated.

### N-terminal sequence analysis

For the deduction of N-terminal sequence, the purified bioactive protein was separated in SDS-PAGE (Bio-Rad Mini-PROTEAN II; 70×100×0.7 mm gels) as recommended by the manufacturer and stained with Coomassie Brilliant Blue R-250. The protein band was transferred to Immobilon-P polyvinylidene (PVDF) membrane (Millipore Corporation, MA, USA) in a transfer buffer containing 10 mM 3-cyclogexylamino-1-propansulfonic acid (CAPS) at a constant current of 350 mA for 60 min in a Mini Trans-Blot® Cell (Bio-Rad Laboratories, Hercules, CA). The membrane was subsequently stained with CBB staining solution and destained. The bands excised from the membrane acted as a source for N-terminal sequence analysis. The sequencing was accomplished by using Procise (pulsed liquid PVDF) 492 protein sequencer (Applied Biosystems, Farmington, MA, USA) using Phenylthiohydantoin (PTH) as standard. BLASTp at NCBI server (http://blast.ncbi.nlm.nih.gov/) was conducted to study the identity of the protein from the sequencing results. Homology search was performed using Swiss-Prot and TrEMBL databases with a BLAST algorithm.

### Construction of degenerate primers and cloning of partial cDNA sequence

The most conserved region of the nucleotide sequence corresponding to the N-terminal protein sequence was the basis for the design of the forward primer and a well-conserved internal sequence region of the most identical proteins acted as the basis for reverse degenerate base primer (Table 1).

**Table 1:** Primers used in the present study.

Primers	Sequence (5′-3′)
Gene specific primer (forward)	CTTCAAGACTTCTGTGTGGC
Gene specific primer-degenerate (reverse)	GC**R**C**SR**GGGTG**H**GTGTG **R-** A+G; **S-** C+G; **H-** A+T+C
5′-GSP1 (reverse)	GGTTCTTGCAGGAGTAGCCTGCAGG
5′-nGSP2 (reverse)	AGAGTAGTCCGCCACACAGAAGTCT
3′-GSP1 (forward)	AATCTCCCTTGCTCGGCCGGACTTA
3′-nGSP2 (forward)	GGTCATCCCTTTCCACACGCACCC
5′-CDS Primer A (Oligo-dT adaptor)	(T)_25_VN (N = A,C,G or T); (V = A,G or C)
3′-CDS Primer A (Oligo-dT adaptor)	AAGCAGTGGTATCAACGCAGAGTAC(T)_30_VN
SMARTer sequence	
SMARTer IIA oligonucleotide	AAGCAGTGGTATCAACGCAGAGTACXXXXX
undisclosed base	
Universal adaptor primer	CTAATACGACTCACTATAGGGCAAGCAGTGGTATCAACGCAGAGT
SMARTer sequence	
M13-F (forward)	GTAAAACGACGGCCAG
M13-R (reverse)	CAGGAAACAGCTATGAC

Total RNA was isolated from young leaves of mulberry (*Morus alba*) using the SV Total RNA isolation system (Promega Corporation, Madison, WI, USA) according to manufacturer's instructions. First strand cDNA was constructed with an Oligo-dT primer using the Superscript III first-strand synthesis system (Invitrogen Corporation, Carlsbad, CA, USA) for Reverse-Transcription PCR (RT-PCR). The cDNA synthesis was conducted at 42°C for 60 min, followed by 94°C for 5 min for RTase inactivation. The synthesized cDNA acted as the template for PCR amplification of the partial gene product using the pre-designed primers. The PCR cycles were performed as follows: denaturation at 94°C for 30 sec, annealing at 48°C for 30 sec and extension at 72°C for 30 sec for 30 cycles in a PTC-200 thermal cycler (MJ Research, GMI, Minnesota, USA). The amplified gene product was extracted from 1% agarose gel and purified using Gel SV protocol (GeneAll, Seoul, Korea).

The purified PCR products were refreshed at 72°C for 15 min. to produce sticky or cohesive ends (A-overhangs) using Taq polymerase (Intron Biotechnologies, Kyungki-do, Korea). The sticky end PCR products were ligated into pCR2.1 TOPO TA cloning vector (Invitrogen Corporation, Carlsbad, CA, USA) and transformed into *E.coliend A* ^−^ strain (DH5α) competent cells and spread into Luria bertani (LB) agar ampicillin plates with X-gal spread (20 mg/ml). Blue-white screening of the transformants was performed and subsequently the white-colonies were sub-cultured in LB broth medium with ampicillin (100 µg/ml). The overnight grown broth culture was used for plasmid isolation and purification (GeneAll, Seoul, Korea). The sequencing was carried out with the M13 forward and reverse primers for the TA vector using 454 genome sequencer-GS FLX+ system (Roche Life Sciences, Branford, CT).

### Full length sequence by rapid amplification of cDNA ends (RACE) PCR

To obtain full-length cDNA of the desired gene, 5′- and 3′-RACE PCR were carried out using SMARTer ™ RACE cDNA amplification kit (Clontech laboratories, CA, USA) according to manufacturer's instructions. 1 µg of total RNA isolated from leaves of mulberry was used to synthesize the 5′- and 3′- RACE- ready cDNA with an oligo-dT adaptor primer. Because of the terminal transferase activity of the SMARTScribe reverse transcriptase used, the first strand cDNAs possess the adaptor primer sequence with both 5′- and 3′- ends. Additionally, SMARTER IIA oligonucleotide was added only to the 5′-RACE ready cDNA synthesis reaction. For 5′-RACE, the first PCR was carried out with the universal primer and gene-specific reverse primer 1 (5′-GSP1), followed by nested PCR with 5′-RACE first product and nested gene specific reverse primer 2 (5′-nGSP2). For 3′-RACE, the first PCR was carried out with the universal primer and gene specific forward primer 1 (3′-GSP1), followed by a nested PCR with 3′-RACE first product and nested gene-specific forward primer 2 (3′-nGSP2). The PCR amplification was done as follows: denaturation at 94°C for 3 min, annealing at 58°C for 30 sec, extension at 72°C for 1 min for 25 cycles. The nested PCR products were extracted and purified from 1% agarose gel by using AccuPrep PCR and Gel purification kit (Bioneer Company, Daejon, Korea) and subsequently cloned into TOPO TA cloning vector (Invitrogen Corporation, Carlsbad, CA, USA) and transformed into competent *E. coli* DH5α cells and sequenced. The sequences of all the primers used are presented in Table 1.

The novel sequence have been submitted to European Nucleotide Archive-European Bioinformatics Institute (ENA-EBI) under the accession number- HE805964.

### DNA and protein sequence analysis

The full-length cDNA sequence and the deduced protein sequence have been analyzed using the *in silico* approaches. Multiple sequence analysis and percent identity matrix of deduced protein sequence of the novel germin-like protein from *M. alba* was done in comparison with other representative plant groups with the aid of Clustal X version 1.83 [34]. The germin and germin-like protein sequences of the representative plant groups were extracted from the GenBank repository at National Centre for Biotechnology Information (NCBI) web-site (http://www.ncbi.nlm.nih.gov/pubmed/) and have been presented in Table 2. The prediction of the putative signal peptide sequence was done at the Signal 4.0 server (www.cbs.dtu.dk). The protein sequence analysis tools used in the study towards the prediction of theoretical MW and isoelectric point (pI) were done at the ExPASy bioinformatics resource portal (http://expasy.org). ProtParam tool at ExPASy was used to compute the various physical and chemical parameters of the deduced protein sequence. PeptideCutter tool at ExPASy was used to predict the potential cleavage sites in the sequence cleaved by proteases. ProtScale tool at ExPASy was used to compute and represent the profile produced by amino acid scale on the protein. The prediction of N-glycosylation sites were confirmed at NetNGlyc 1.0 server (http://www.cbs.dtu.dk/services/NetNGlyc/). TargetP 1.1 server at cbs.dtu.dk was used to predict the cleavage site and possible localization of the protein [35]. Post-translational modifications as N-acetylation, O-glycosylation, phosphorylation and kinase-specific phosphorylation were also predicted at cbs.dtu.dk with the aid of NetAcet 1.0, NetOGlyc, NetPhos 2.0 and NetPhosK 1.0 server respectively [36]–[39]. Disulfide bonds were predicted by the Cys_REC tool (version 2.0) from Softberry (http://linux1.softberry.com/berry.phtml/). The superfamily and the conserved domains including the metal binding sites were predicted using the ScanProsite tool (http://prosite.expasy.org/scanprosite/) and the InterPro Scan (version 4.8) at European Bioinformatics Institute (http://www.ebi.ac.uk/tools/pfa/iprscan/). ORF and protein statistics were inferred by the EditSeq tool of Lasergene 9.0 software of DNASTAR program (http://www.dnastar.com/). The software was also used to study the codon usage, base composition in the ORF and predicted structural class of the whole and mature protein including the chemical formula.

**Table 2:** Accession number of germins and germin-like proteins from representative plant groups.

S. No.	Species	Abbreviation	Group	Accession No.
1	*Pisum sativum*	Ps-GER1	Germin-like protein	CAB65369
2	*Vitis vinifera*	Vv-Glp2	Germin-like protein	ABH09468
3	*Nicotiana plumbaginifolia*	Np-NEC1	Germin-like protein	Q9SPV5
4	*Arabidopsis thaliana*	At-Glp5	Germin-like protein	AAB51569
5	*Hordeum vulgare*	Hv-GER5a	Germin-like protein	ABG46237
6	*Hordeum vulgare*	Hv-GER1a	True Germin	ABG46232
7	*Triticum aestivum*	Ta-GER2	True Germin	P15290
8	*Triticum aestivum*	Ta-GER3	True Germin	P26759
9	*Lolium perenne*	Lp-OXO1	True Germin	CAC19429
10	*Oryza sativa*	Os-GF2	True Germin	ABF988325
11	*Vigna unguiculata*	Bu-Glp	Germin-like protein	BAC53790
12	*Physcomitrella patens*	Pp-Glp3a	Germin-like protein	BAD86499
13	*Physcomitrella patens*	Pp-Glp6	Germin-like protein	BAD86502
14	*Arabidopsis thaliana*	At-Glp6	Germin-like protein	P92997
15	*Vitis vinifera*	Vv-Glp3	Germin-like protein	AAQ63185
16	*Hordeum vulgare*	Hv-GER3a	Germin-like protein	ABG46234
17	*Hordeum vulgare*	Hv-GER4d	Germin-like protein	ABG46236
18	*Atriplex lentiformis*	Al-Glp	Germin-like protein	BAA78563
19	*Prunus persica*	Pp-ABP20	Germin-like protein	U81162
20	*Prunus salicina*	Ps-Glp2	Germin-like protein	EU310512
21	*Prunus persica*	Pp-ABP19	Germin-like protein	U79114
22	*Prunus salicina*	Ps-Glp1	Germin-like protein	EU310513
23	*Vitis vinifera*	Vv-Glp6	Germin-like protein	ABL60875
24	*Gossypium hirsutum*	Gh-Glp1	Germin-like protein	AA092740
25	*Sinapis alba*	Sa-Glp	Germin-like protein	P45854
26	*Arabidopsis thaliana*	At-Glp3a	Germin-like protein	P94072
27	*Hordeum vulgare*	Hv-GER2a	Germin-like protein	ABG46233
28	*Prunus nil*	Pn-Glp	Germin-like protein	P45853

The amino acid sequences were used for the multiple sequence alignment, construction of percent identity matrix by Clustal X (version 1.83) and phylogenetic analysis by MEGA 5.05 (version 5.05).

### Phylogenetic analysis

Prior to phylogenetic analysis, Clustal X software (version 1.83) was used to perform multiple sequence alignment of the deduced amino acid sequence of the novel germin-like protein of *M. alba* with other true germin and germin-like sequences from representative plant groups. MEGA 5.05 [40] software was used to construct the consensus phylogenetic tree using the unweighted pair group method with arithmetic mean (UPGMA) method [41]. To evaluate the branch strength of the phylogenetic tree, bootstrap consensus tree inferred from 5000 replicates were taken for analysis.

### Secondary structure prediction analysis

Secondary structure predictions were performed using the consensus prediction programme available at PSIPRED protein structure prediction server 2.6. (http://bioinf.cs.ucl.ac.uk/psipred/). The generated consensus leads to three possible states for each residue (“H”: alpha helix, “E”: Extended strand and “C”: Coil). The accuracy of prediction currently may reach a score of 80.7%.

The homology modeling was performed at SWISS MODEL workspace (http://swissmodel.expasy.org/workspace) and a theoretical model was predicted for the novel germin-like protein from *M. alba* based on template (1fi2A at 1.60 A resolution) belonging to the oxalate oxidase germin. The predicted structure with QMean score 4 of 0.5 was visualized in RasWin Molecular Graphics Version 2.7.5 (http://www.rasmol.org/). The local model quality estimation was done with Anolea and QMean graphics under the workspace.

## Results and Discussion

### Isolation and purification of novel protein from silkworm fecal matter

**Figure 1:**
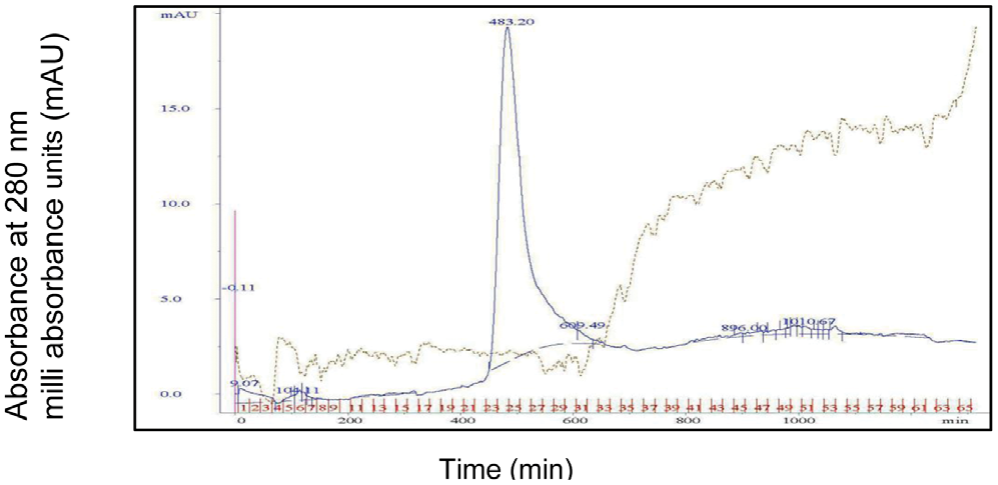
Gel filtration chromatography profile of bioactive peak ‘A’ fraction. The column purified dialysate was subjected to Gel filtration chromatography using Sephadex G-75 column (1.6×70 cm) at a flow rate of 0.1 ml/min and sample volume of 500 µl. Protein was eluted with 0.02 M phosphate buffer solution at pH 7.4. The retention time of the peak was 483.2 min with an area of 899.6 mAU*min and a peak area of 90.5%.

**Figure 2:**
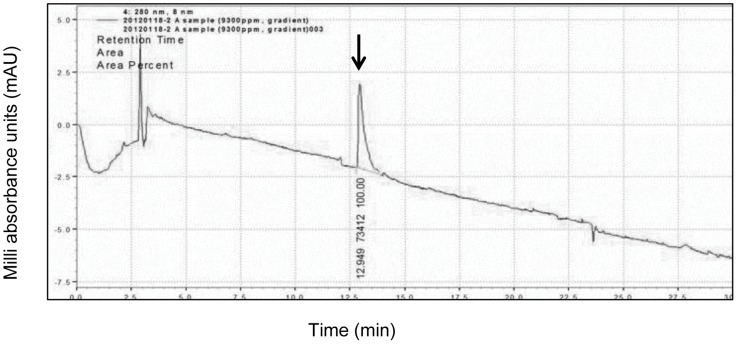
Analytical HPLC profile of purified novel protein found to have antibacterial activity. Peak ‘A’ fractions obtained from Gel filtration chromatography was analyzed using the reverse-phase C18 column (4.6×250 mm) and eluted with 30–70% acetonitrile – 0.05% TFA linear gradient mode with a flow rate of 1.0 ml/min and detection wavelength of 280 nm. A single peak (denoted by arrow) with a retention time of 12.95 min was observed. The area percent for the peak was 100 and area was 73412.

The silkworm fecal matter is one of the rich and promising sources for exploring proteins having potent antimicrobial and antiviral functions. The present study explored the thermally-resistant bioactive protein components of silkworm fecal resources as the extraction process was accomplished at an elevated temperature of 60°C. Novel bioactive proteins were subsequently purified by conventional biochemical techniques, such as 40% ammonium sulphate precipitation, silica column chromatography and GFC with Sephadex G-75. GFC with Sephadex G-75 resin as matrix resolved the column chromatography purified fractions into two peaks A and B. Peak A was resolved as a sharper peak, whereas peak B was observed as a broader peak and assumed to contain low molecular weight proteins/peptides. The UV spectrum plot was recorded at absorption of 280 nm. The peak A fraction had a retention time and volume of 483 min and 43 ml. respectively as observed in AKTA Prime Unicorn 5.0 software (Fig. 1). Subsequently, the peak A fractions were characterized by denaturing and non-denaturing gel systems and tested for its positive activity against some pathogenic microbes (bacteria and fungi).To check the homogeneity of the bioactive peak fraction obtained from GFC, the above sample was lyophilized and subjected for HPLC using reverse- phase C18 column. A prominent peak (Fig. 2) at a retention time of 12.95 min. was observed that was purified using preparatory column for HPLC. Analytical-scale HPLC is always the final step to check for the purity of the already prepared and characterized proteins and have been consistently used in most of the earlier referred reports.

The proteins resolved through the above methodology seemed appropriate, though it was understood that use of analytical GFC columns as Superdex or superose may be necessary for well-resolved peaks. Also the resolving range can be extended to include much bigger proteins as well. Earlier reports of using similar strategy conformed to the results of the present study as two well-resolved peaks were observed with functional activity of protein in one of the peaks [13], [42]. A report of partial purification of a novel bioactive protein component is also available, that has used adsorption chromatography using silica gel mesh to resolve silkworm fecal extract into two peaks, with one of them reported to have strong antimicrobial and analgesic activity [18]. In all the above reports, the extraction was accomplished at a temperature of 50–60°C, to exploit the thermally resistant proteins, but the proteins identified seems to be different. It therefore, needs to be foretold that, silkworm fecal matter is a natural, exploitable resource for the discovery of potential molecules of significant pharmaceutical use. Some researchers have exploited the antimicrobial RFP present in silkworm excreta, by preparing the extract at 4°C with 20% solid ammonium sulphate precipitation and GFC with Sepharose-6B. The red fluorescence of the protein band was observed at 366 nm [26]. Antiviral proteins have been purified from silkworm gut juice using similar strategies of 40% ammonium sulphate precipitation and further purification by GFC [1], [11].

**Figure 3:**
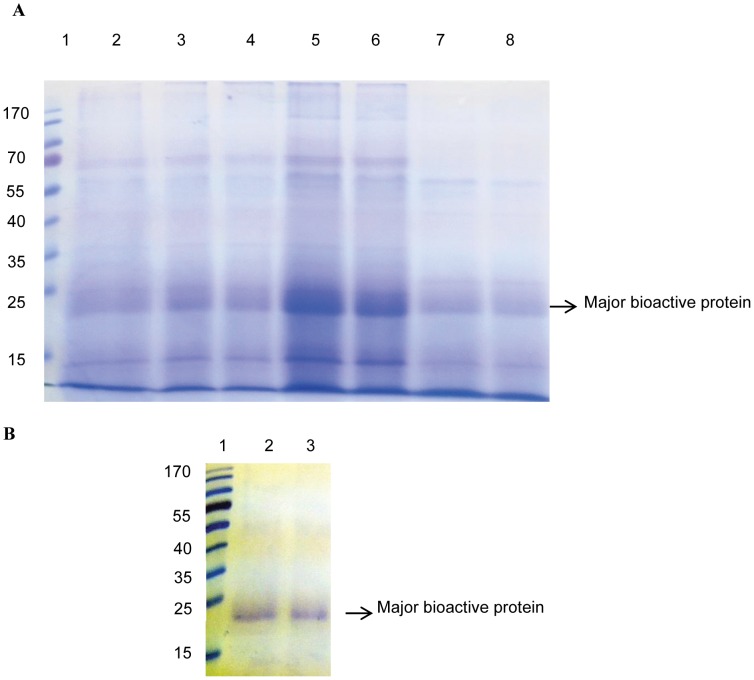
A. SDS-PAGE analysis of saturated ammonium sulphate precipitated proteins. The extracts from silkworm fecal matter was subjected to saturated ammonium sulphate cuts at 20, 40 and 60% and dialyzed against 0.02 M PBS, pH 7.4 for duration of 48 hrs and run on 12% reducing gel and subsequently stained with Coomassie brilliant blue R-250. Lane 1- Standard molecular weight protein marker (Fermentas, Thermo Fisher Scientific, Maryland, USA), Lane 2- Non-dialyzed sample, Lane 3 and 4- 20% cut, Lane 5 and 6- 40% cut, Lane 7 and 8- 60% cut. **B. SDS-PAGE of purified bioactive protein.** The gel-filtered fractions were pooled and concentrated by lyophilization. The lyophilized protein was mixed in an appropriate volume of 0.02 M PBS, pH 7.4 and run on 12% polyacrylamide gel and subsequently stained with Coomassie brilliant blue R-250. Lane 1- Standard molecular weight protein marker (Fermentas, Thermo Fisher Scientific, Maryland, USA), Lane 2 and 3- Purified bioactive protein.

Ammonium sulphate precipitation of proteins and optimization of percent saturation that gives the maximum concentration of the target protein is a key to such extraction steps. In the present study, a series of saturated ammonium sulphate cuts were used to precipitate the proteins from the fecal extract (Fig. 3A). Many discretely sized polypeptides exhibiting a wide range of molecular masses were present in fecal extracts. The most abundant polypeptide was strongly concentrated at about 40% saturated ammonium sulphate and also significantly the peak A fraction in GFC were stronger at lower cuts, whereas the peak B fractions were stronger at about 60% cut. Report of partial purification of RFP from silkworm gut juice involved the use of 40% ammonium sulphate saturation, followed with dialysis and separation by native PAGE [7], [43]. In another attempt, processed threonine deaminase (pTD2) was purified from *M. sexta* feces by precipitating the enzyme at 65% ammonium sulphate cut, followed by DEAE-cellulose chromatography and Superose-12 gel filtration chromatography [76].

The peak A fractions routinely collected from GFC was pooled, calculated for protein concentration and run down in SDS-PAGE under reducing conditions. The coomassie staining of the gel showed the conspicuous presence of a single polypeptide of approximately 21.5 kDa (Fig. 3B), inferred through standard protein molecular weight markers. The polypeptide purified by chromatographic separations was the most abundant protein that was visualized after extraction, 40% ammonium sulphate precipitation and dialysis, though additional polypeptides were also observed. It may be presumed, at this point that these additional polypeptides may correspond to proteins expressed during plant defense against insect herbivores or pathogenic stresses. A protein of ∼35 kDa and two protein bands of about 23 and 16 kDa from non-reducing gel electrophoresis have been reported before from silkworm fecal matter that were bound to carbohydrates and was believed to be of plant origin[13], [42]. This robust approach of screening the silkworm fecal extract may provide insights into the evolution of host mulberry proteins implicated in defense.

### Antimicrobial and antiviral activity of the novel protein

**Figure 4:**
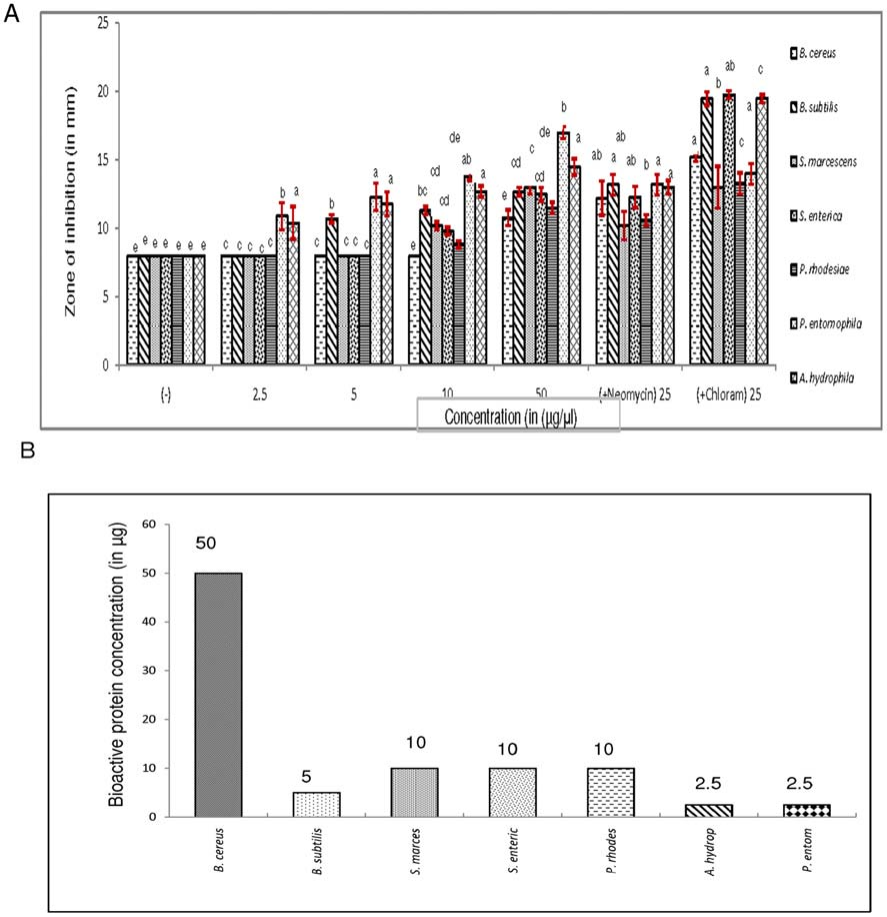
A. Antibacterial activity of purified novel protein (at various concentrations) from silkworm fecal extract. The extract was prepared in 0.02 M PBS and purified by the process of 40% ammonium sulphate precipitation, dialysis, gel-filtration chromatography and RP-HPLC. **B.Minimum inhibitory concentrations of purified novel protein.** Anti-bacterial activity was tested against Gram (+ve) bacterial strains such as *Bacillus cereus* B33 (NCBI number FJ483513), *Bacillus subtilis* EG1 (NCBI number FJ483514) and Gram (−) bacterial strains as *Serratia marcescens* PRC-5 (NCBI number JN816402), *Salmonella enterica* KACC 10763 (serovar enteridis), *Pseudomonas rhodesiae* NO5 (NCBI number FJ462694), *Pseudomonas entomophila* MG23S and *Aeromonas hydrophila* AKR 1 (NCBI number FJ462702). Bars show the diameter of zone of inhibition (mean of three replicates) in millimeter ± standard error. Standard antibiotics, namely, Neomycin and Chloramphenicol (25 µg) were used as positive controls. 8 mm is the diameter of bore prepared. No inhibition zones were observed for negative control (0.02 M PBS, pH 7.4). Figures on the top of the bar represent the minimum concentration at which zone of inhibition was noticed.

The purified novel protein was studied for its activity against some Gram (+) bacteria as *B. cereus* and *B. subtilis* and Gram (−) bacteria as *S. marcescens*, *S. enterica*, *P. rhodesiae*, *P. entomophila* and *A. hydrophila* (Fig. 4A). The purified protein from silkworm fecal extract showed concentration dependent activity against all the bacterial strains, measured by the produced zone of inhibition in agar plates. The significant value of antibacterial activity of the novel protein has also been compared to standard antibiotics as Neomycin and Chloramphenicol at a concentration of 25 µg and buffer control. At a lower concentration of 2.5 µg, greater zone of inhibition was observed against Gram (−) *P. entomophila* followed by *A. hydrophila*. At 5.0 µg concentration of the purified protein, activity was marked against Gram (+), *B. subtilis* and at 10.0 µg, activity was observed against other Gram (−) bacteria tested as *S. marcescens*, *S. enterica* and *P. rhodesiae*. Gram (+) bacterial strain, *Bacillus cereus* was able to strongly counteract the active principles of the novel protein as zone of inhibition was only observed at about 50 µg dose of the protein. No clear zones of inhibition was evident below 2.5 µg concentration of the protein, suggesting it to be the minimum inhibitory concentration for some Gram (−) bacterial strains. Chloramphenicol as an antibiotic was found to be more efficient in comparison to Neomycin as observed with the greater zones of inhibition against the bacterial strains tested.

The results of the MIC studies are presented in Fig. 4B. The MIC of the purified protein was low against most of the bacterial strains tested as 2.5 µg, 5 µg and 10 µg of the purified protein was sufficient to inhibit considerable proliferation of *P. entomophila* and *A. hydrophila*, *B. subtilis* and *S. marcescens*, *S. enterica* and *P. rhodesiae* respectively. The SE-RFP reported earlier also showed growth inhibition of pathogenic bacterial strains and the MIC values were in the range of 7.5–25 µg/ml. The SE-RFP activity was high against bacterial strains as *S. aureus*, *K. pneumoniae* and *E.coli* [25]. In another report of purification of ∼35 kDa protein from fecal extract, antibacterial activity was observed at 50 µg and the MIC against clinical strains as *S. hemolyticus*, *S. aureus*, *S. typhi*, *P. aeruginosa*, *B. subtilis* and *P. aeruginosa* were observed at 30 µg.

**Figure 5:**
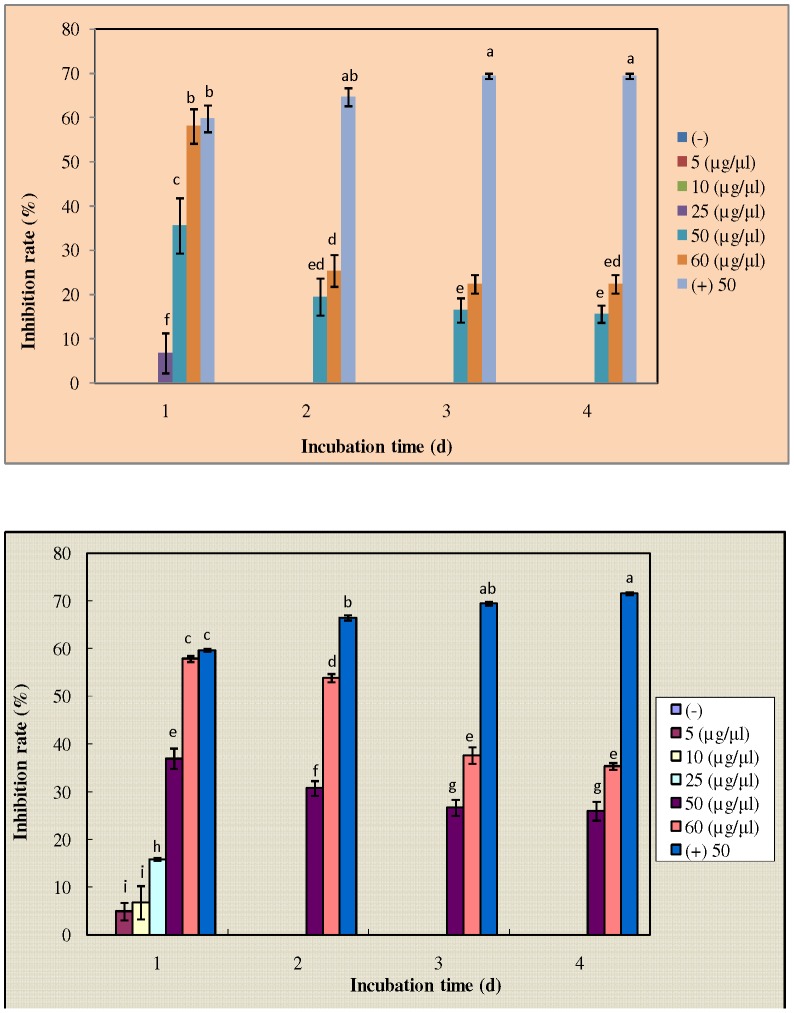
Antifungal activity of purified novel protein. The silkworm fecal matter extract was prepared in 0.02 M PBS and purified by the process of 40% ammonium sulphate precipitation, dialysis, gel-filtration chromatography and RP-HPLC. (A) The activity of purified novel protein at concentrations of 5, 10, 25, 50 and 60 µg/µl against *F. solani* strain (KACC 40384). (B) The activity of purified novel protein at concentrations of 5, 10, 25, 50 and 60 µg/µl against *F. oxysporum* strain (KACC 40032). Bars show IR% (mean of three replicates) ± standard error. Cycloheximide at a concentration of 50 µg/µl acted as positive control. The results were statistically analyzed by one-way Anova and Tukey's multiple range test at 95% confidence (*P<0.05*). Different subscripts present significant differences within and among groups.

**Figure 6:**
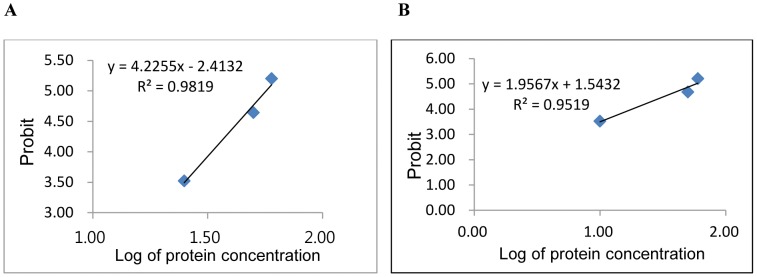
Antifungal activity of purified novel protein. Relationship between inhibition probability and protein concentration logarithm for (A) *F. solani* and (B) *F. oxysporum*. The IR % of the purified protein against *Fusarium* species was converted to probit scale (Table 3). The probit of growth inhibition and log of protein concentration was plotted to calculate IC_50_ values from the correlation coefficients obtained.

The results of antifungal assays of the purified proteins as shown in Fig. 5 indicated high antifungal activity against fungal strains as *F. solani* and *F. oxysporum*. The observed activity against *F. solani* (Fig. 5A)was recorded at a protein concentration of 50 and 60 µg/µl, even though with the increase in incubation time the activity was reduced.. There was a significant increase in activity at 1 day incubation, followed with a significant decline at the 2^nd^ day followed with stable maintenance of activity after 2 days. The pattern was similar with *F. oxysporum* (Fig. 5B), though the significant decline in activity was stabilized after 3 days of incubation with the purified protein. At a lower concentration of the protein, the activity was not marked with the increase in incubation time. The inhibitory concentration mean values were changed into the probit scale (Table 3) and were plotted against log of protein concentration to study the correlation and deduce the IC_50_ values (Fig. 6). The IC_50_ or effective concentration (EC_50_) values for *F. solani* and *F. oxysporum* was 56.8 and 58.43 µg/µl respectively. Antifungal assay for SE-RFP showed a good activity against *C. albicans* and *A. flavus*, whereas activity was low against *A. niger* as observed from the MIC values.

**Table 3:** Activity of purified protein against fungal *Fusarium* solani (KACC 40384) and *Fusarium oxysporum* (KACC 40032) species.

*Fusarium solani* KACC 40384					*Fusarium oxysporum* KACC 40032				
Sam. Conc.(µg/µl)	LOG	Rep.	IR %	Probit	Sam. Conc. (µg/µl)	LOG	Rep.	IR %	Probit
5	0.70	1	0.00		5	0.70	1	4.05	3.36
		2	0.00				2	7.19	
		3	0.00				3	3.86	
		mean	0.00				mean	5.03	
		S.E.	0.00				S.E.	1.53	
10	1.00	1	0.00		10	1.00	1	7.90	3.52
		2	0.00				2	9.63	
		3	0.00				3	2.89	
		mean	0.00				mean	6.81	
		S.E.	0.00				S.E.	2.86	
25	1.40	1	1.50	3.52	25	1.40	1	15.61	4.01
		2	9.55				2	16.18	
		3	9.37				3	15.99	
		mean	6.81				mean	15.93	
		S.E.	3.75				S.E.	0.24	
50	1.70	1	37.75	4.64	50	1.70	1	37.38	4.67
		2	40.45				2	34.49	
		3	28.57				3	38.54	
		mean	35.59				mean	36.80	
		S.E.	5.08				S.E.	1.70	
60	1.78	1	58.72	5.20	60	1.78	1	58.57	5.20
		2	61.59				2	57.80	
		3	52.86				3	57.42	
		mean	58.06				mean	57.93	
		S.E.	3.19				S.E.	0.48	

Inhibition rate % was converted to probits by looking up the corresponding % in Finney's table.

**Figure 7:**
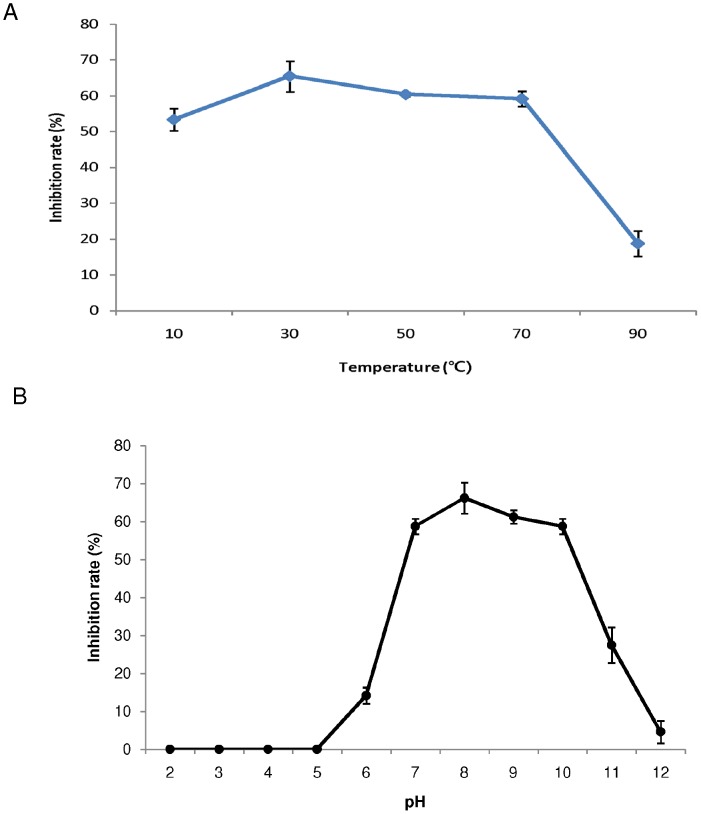
Biochemical properties of purified novel protein. (A) Temperature-dependent assay of purified novel protein. (B) pH-dependent assay of purified novel protein. The activity was assayed against bacterial strain, *Bacillus subtilis* by calculating the IR% from the visible zone of inhibition at the indicated temperatures (°C) and in the following buffer systems: Gly-TrisHCl (pH 2.0–3.0), sodium acetate (pH 4.0–5.0), sodium phosphate (pH 6.0–7.0), Tris-HCl (pH 8.0–9.0) and sodium carbonate (pH 10.0–12.0).

The purified novel protein extracted from silkworm fecal extract at 60°C, showed potent antibacterial activity at most of the temperatures tested and was considered to be thermally stable. This finding will be significant as previous work have not reported such character of the extracted proteins. The proteins extracted previously showed a decrease in their activity when incubated below 10°C or above 70°C and also showed activity in the pH range of 6.5–8.5 [13], [42]. Ma-Glp was also active over a wide range of temperatures with an anti-bacterial inhibition rate (against *B. subtilis*) of 50–70% at temperatures ranging from 30–70°C (Fig. 7A). In addition, it showed high inhibition rate in an alkaline pH range that matches that of the lepidopteran midgut, and little or no activity was observed at pH below 6.0 (Fig. 7B).

### Mass Spectrometry analysis and Edman sequencing

**Figure 8:**
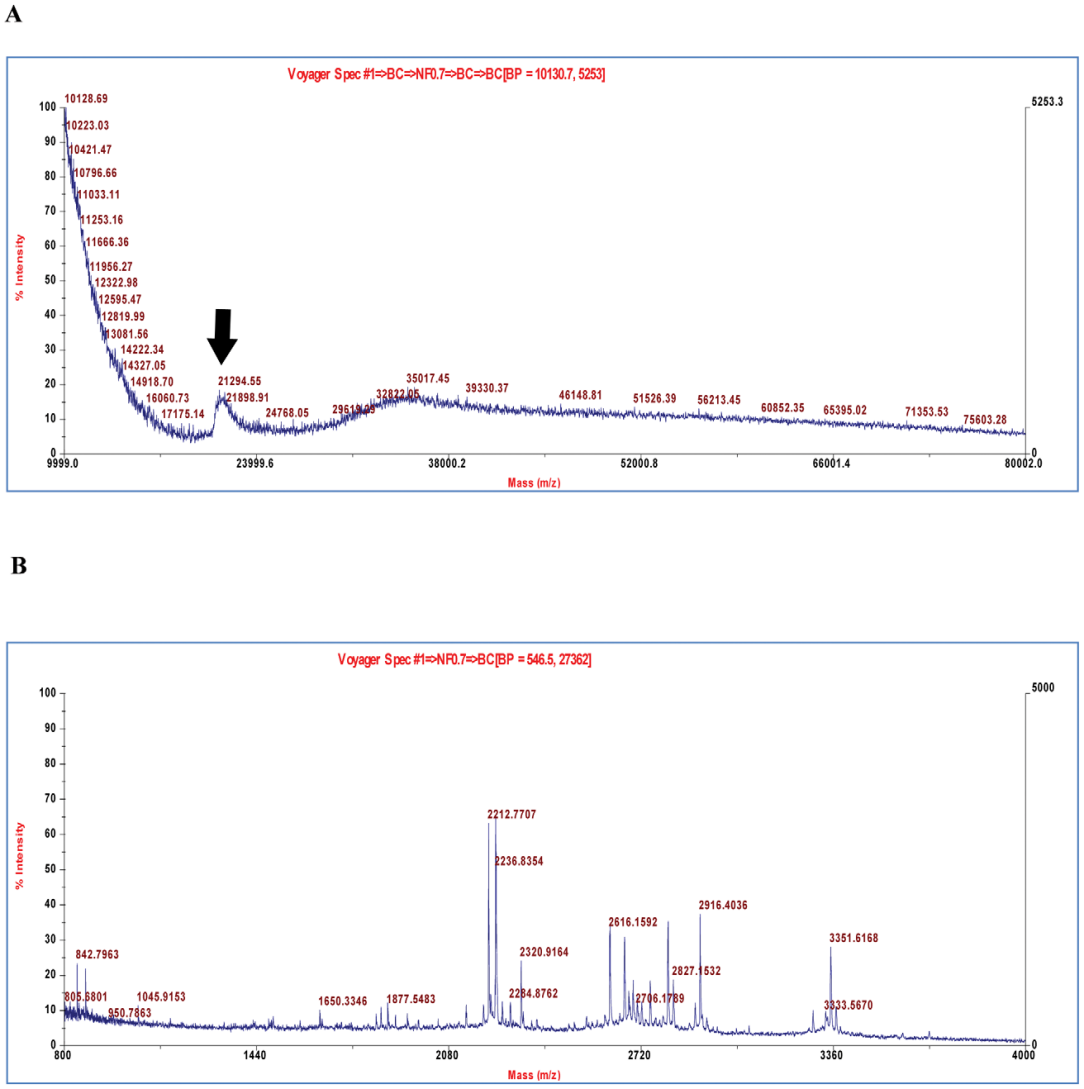
MALDI-TOF MS analysis of the purified novel protein. (A) Determination of intact molecular mass using Sinapinic acid as matrix. Protein samples were cleaned using the ZipTip ™ C18 resin. The scan range between 10,000–80,000 Da is presented with the singly charged peak [M+H]^+^for the target protein is depicted by arrow. (B) *In-gel* tryptic digestion spectra of the protein using α-cyano-4-hydroxycinnamic acid (CHCA) as matrix. The scan range between 800–4000 Da is presented with the appropriate masses of generated peptides is highlighted on the top of the peak. In-gel tryptic map of BSA in CHCA matrix was used as the internal calibrated standard.

The completely purified protein from silkworm fecal extract was subjected to MALDI-TOF-MS for determination of intact molecular mass and subsequently the in-gel separated proteins were digested with trypsin to generate the peptide fragments towards protein identification. MALDI-TOF-MS analysis of purified protein showed a molecular mass of 21,285 Da (Fig. 8A), which was in good agreement with the size observed by SDS-PAGE. The *m/z* values for in-gel digested peptide fragments from the purified protein was generated using MALDI-TOF-MS (Fig. 8B) and was queried for significant match using the available public databases. The mass of the peptide fragments generated did not conform to any significant matches within the database. It is assumed here that the covalent modifications of the polypeptides in the insect gut may prevent protein identification by MS. This was significant towards progress of our attempts to characterize and identify the protein by Edman sequencing.

**Figure 9:**
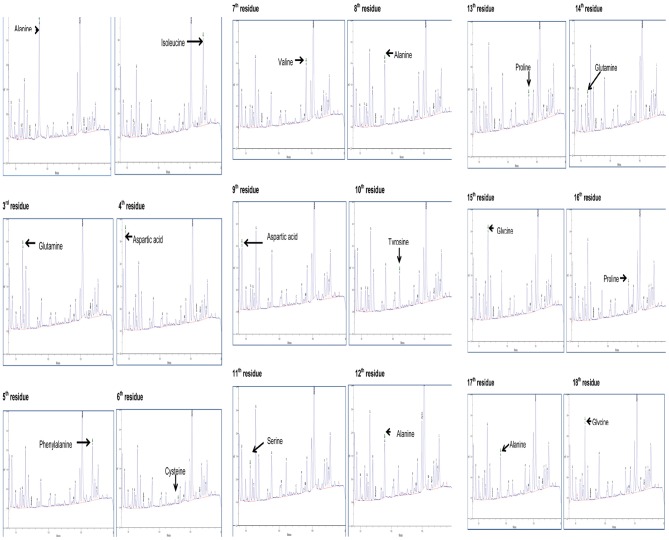
N-terminal sequencing of the purified novel protein. The purified protein was transferred onto an Immobilon-P (PVDF) membrane, excised and sequenced using a PROCISE (pulsed liquid PVDF) Applied Biosystems sequencer using PTH as standard. The N-terminal 18 amino acids were sequenced and subsequently BLASTp was conducted to identify the protein.

For N-terminal sequencing by Edman degradation method, the proteins separated in denaturing-gel was transferred to Immobiline-P PVDF membrane and sequenced. The first 18 amino acid residues confirmed through the sequencing were AIQDFCVADYSAPQGPAG (Fig. 9) and was predicted to show homology with Auxin-binding proteins and proteins from the germin class. Since the predicted homology was targeted towards the plant-based proteins and silkworm feeds on mulberry, an exhaustive survey was conducted to find similar proteins in the food source. It was found that similar proteins have not been earlier known in mulberry. The ∼35 kDa protein from silkworm fecal extract reported before had shown homology with DEAD-box-ATP-dependent RNA helicase 45 by MALDI-TOF-MS analysis and NCBI database search [42]. In variance to our report, the N-terminal sequence of SE-RFP had shown sequence alignment with *Bombyx mori* proteins [25]. The predicted novelty of the protein was a significant highlight of the present study, which led us to study the nucleotide sequence and its analysis in detail.

### cDNA cloning and full-length nucleotide sequence

**Figure 10:**
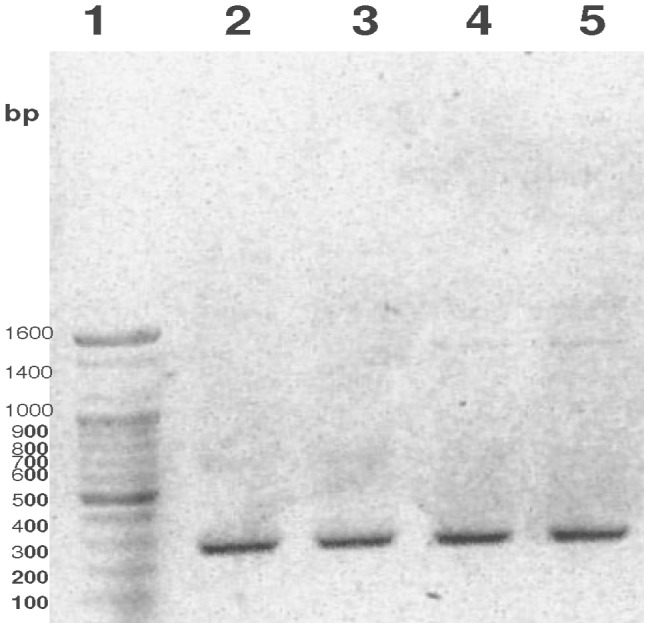
Cloning of partial cDNA sequence from *M. alba* leaves. The N-terminal sequence of the novel protein was subjected to BLASTp to get its homology with the most identical sequences. The conserved N-terminal nucleotide sequence acted as forward primer and degenerate primers constructed from the internal conserved sequences acted as reverse primer to amplify and clone the prepared cDNA corresponding to the novel gene into TOPO TA vector. The construct (vector carrying the partial cDNA insert) was transformed into DH5α strain of *E. coli* and sequenced. The expected size of the partial cDNA was 270 bp. Lane 1- 100 bp DNA ladder (Bioneer, Daejeon, Korea); Lane 2 and 3- Clones from 100 µl spread; Lane 4 and 5- Clones from 250 µl spread.

To decipher the nucleotide sequence of the novel protein purified and characterized from silkworm fecal extract showing homology with plant-based protein, we isolated total RNA from mulberry (*M. alba*; white mulberry) leaves and synthesized cDNA towards amplification of the target gene using degenerate primers. The N-terminal protein and corresponding nucleotide sequence was conserved among the proteins showing homology and was therefore designed as the forward primer whereas an internal conserved sequence was targeted to act as reverse degenerate primer towards amplification of the desired target gene. The partial cDNA product thus was expected to produce a PCR fragment of approximately 270 bp. The amplified PCR product acted as the insert and was ligated and cloned into TOPO TA cloning vector and subsequently transformed into competent *E.coli* (DH5α) cells. The transformants were screened, subcloned, and plasmids with the insert run on agarose gel to confirm the expected size (Fig. 10).

**Figure 11:**
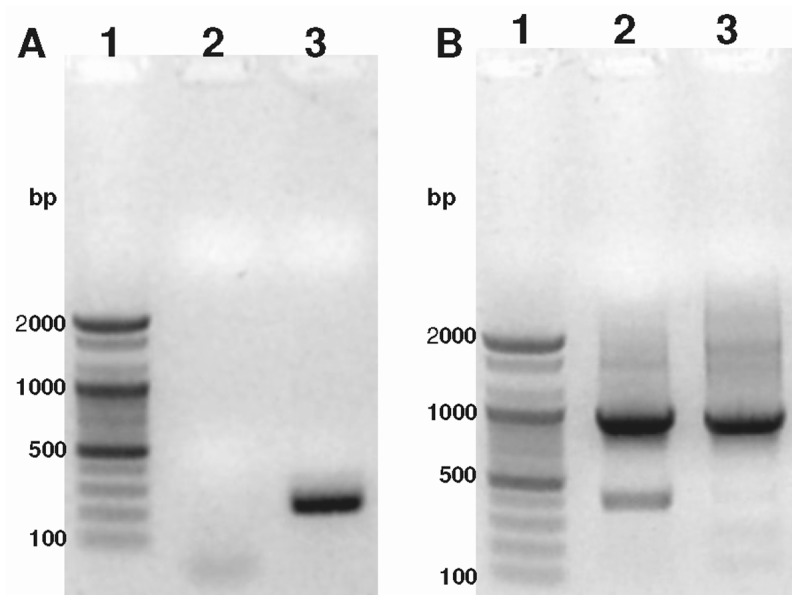
Full-length sequence of novel gene by 5′- and 3′-RACE-PCR. Total RNA was extracted from *M. alba*, followed with the generation of 5′- and 3′-RACE ready cDNA The RACE ready cDNAs were amplified using gene-specific primers constructed from the partial cDNA sequence in two PCR reactions (first PCR followed with nested PCR) using universal primer mix. The nested PCR products (both 5′- and 3′-products were cloned in TOPO TA vector and sequenced. (A) 5′-RACE product. (B) 3′-RACE product. Lane-1- 100 bp DNA ladder (Bioneer, Daejeon, Korea); Lane 2- first PCR product with GSP-1; Lane 3- nested PCR product with GSP2.

The partial cDNA sequence was the basis for the design of primers towards the elucidation of full-length gene sequence by 5′- and 3′-RACE-PCR (Fig. 11). The method used the total RNA extracted from *M. alba* leaves to construct 5′- and 3′-RACE ready cDNA and two simultaneous PCR reactions (first PCR and nested PCR), followed with cloning into TA vector to get the full-length sequence. The full length sequence has been registered with EBI-ENA under the accession no. HE805964.

The novel full-length cDNA corresponded to germin-like protein class of proteins and was the first report of the same from *M. alba*.

### Sequence analysis and characterization of germin-like protein from *M. alba*

The full-length germin-like protein cDNA isolated from M. *alba* was observed to have a N-terminal sequence that was similar to the characterized sequence of the purified protein extracted from silkworm fecal matter. This makes it clear that the major protein purified from silkworm fecal matter is the *M. alba* germin-like protein. This marks a significant strategy towards exploiting the fecal resources towards identification and characterization of novel defense proteins of plant origin.

**Figure 12:**
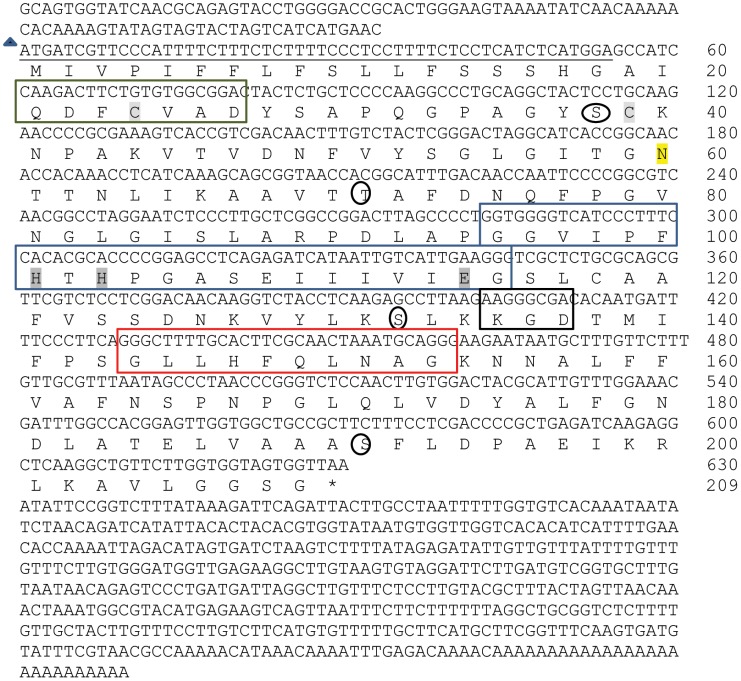
Nucleotide and deduced amino acid sequence of novel germin-like protein from *M. alba*. The putative signal peptide region is underlined. The N-terminal sequence of the novel bioactive protein from silkworm fecal matter corresponds to the sequence following the signal peptide. The three conserved germin/Glp motifs are Glp Box A (Green), Glp Box B (Blue) and Glp Box C (red). RGD-like tripeptides (KGD) are boxed (black). Blue triangle –Initiation codon; * - stop codon; Putative phosphorylation sites are encircled. Potential N-glycosylation is indicated yellow shade. The conserved cysteine residues are shaded. The three histidines and the glutamate amino acid residues involved in metal binding are dark grey shaded. EBI-ENA accession number of *M. alba* germin-like protein is HE805964.

**Figure 13:**
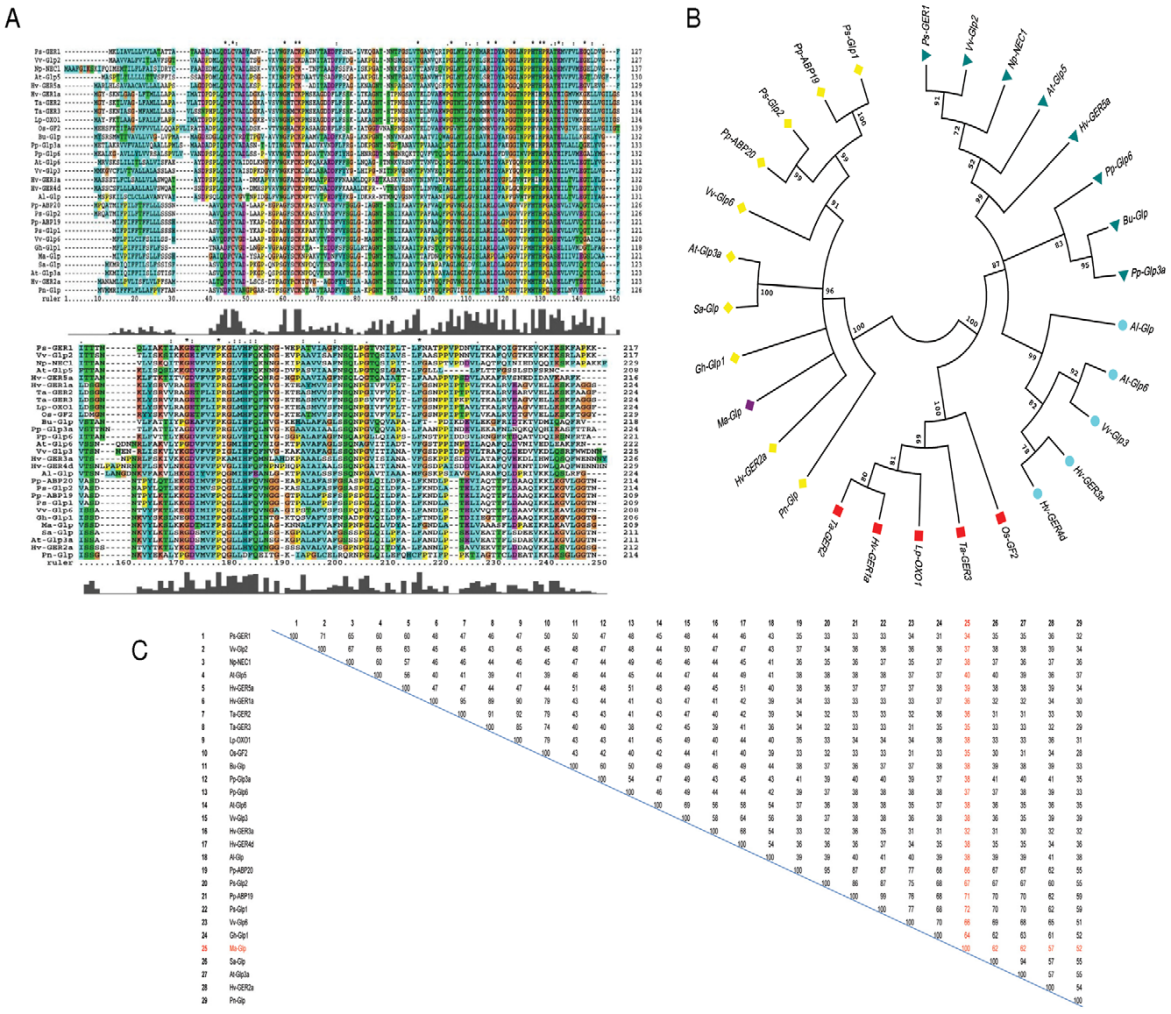
Primary sequence analysis of novel germin-like protein from *M. alba*. (A) Multiple alignment of the deduced amino acid sequence of Ma-Glp with true germins and Glps from other representative plant groups as presented in Table 2. Residues shared by several germins and Glps are indicated with the following symbols: ‘*’-denotes identical residues in all sequences; ‘:’- conserved substitutions according to similar properties of amino acids; ‘.’- semi-conserved substitutions. Dashes indicate gaps used to maximize the alignment. (B) Relationships between members of several species presented as a phylogenetic tree andin ferred using the UPGMA method. The percentage of replicate trees in which the associated taxa clustered together in bootstrap test (5000 replicates) is shown next to branches. Evolutionary analysis was conducted in MEGA 5.0. Circular representation of tree. Cones- Glp subfamily III; Triangle-Glp subfamily II; Circle- Glp subfamily I; Square- True germins (C) Percent identity matrix of the representative species as inferred using Clustal X 1.83.

**Figure 14:**
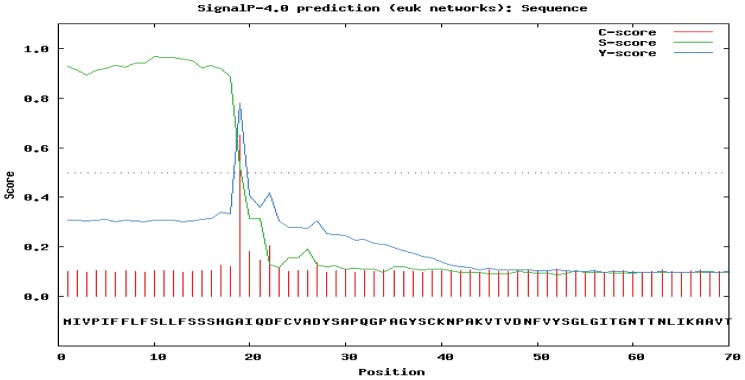
Sequence analysis of novel germin-like protein from *M. alba*. (A) Prediction of putative signal peptide in the sequence by Signal 4.0 server.

The full-length sequence of Ma-Glp was found to comprise of 1238 nucleotides with a cDNA sequence of 630 bp. The cDNA encodes a protein of 209 amino acid residues. The 5′-, 3′- non-coding and poly (A^+^) sequences were 93, 515 and 27 bp, respectively (Fig. 12). Multiple alignments of Ma-GLP sequences with other reported true germin/Glp genes (Fig. 13A) highlighted number of conserved motifs and structural similarities that are common to the plant Glp subfamily [44]. The deduced amino acid sequence of Ma-Glp comprised of a conserved extracellular targeting signal peptide (Fig. 14) located at the N-terminus that is a characteristic feature of the Glp gene family with the exception of *Arachis hypogea* Glp7, predicted to contain a non-cleavable amino-terminal sequence. The lack of a KDEL consensus motif in the sequence targets the protein into the secretory pathway rather than endoplasmic reticulum retention. The signal peptide is predicted to be cleaved between amino acid residues 18 and 19 and the 19^th^ residue acts as the first residue of the mature secreted protein [45], [46]. The mature protein without the putative signal peptide region was found to encode 191 amino acid residues. It is therefore significant here to point out that the bioactive protein purified from silkworm fecal resource is the mature protein or the secreted product of the food plant, *M. alba*.

The most significant attribute of Ma-Glp was that the predicted protein sequence contained the three highly conserved germin/Glp oligopeptides characteristic of Glps [44]. Glp box-A is a highly conserved sequence (QDFCVAD) at the N-terminal site and includes a cysteine residue at position 24 which is followed by a second cysteine at position 39 and may be believed to form an intramonomeric disulphide bridge of the extracellular domain [47]. Glp box-B (G-P-H-HPGASEXXXXX-G) corresponding to amino acid residues from 96–109) is conserved at the internal position of the full-length sequence and Box-C is (GXXHFQXN-G) is conserved at the C-terminal domain, in which X corresponds to any hydrophobic amino acid residue. Glp box-B contains the two histidine and one glutamate residue and box-C contains the third histidine residue involved in heavy metal ion-binding site [48], [49], and considered to be the ligand binding conserved sequence in Auxin-binding proteins/germins class. Therefore, the germin-family signature for Ma-Glp is predicted to be box-B (GVIPFHTHPGASEI). Consistent with Glp from other plant species, a single potential N-glycosylation site (NTTN) was identified in the cDNA sequence with asparagine at position 60 involved in the glycosylation process. Glycosylation in germins appears to be essential in protein-protein interactions but not for enzyme activity [50]. The RGD-like tripeptides (KGD) motif sequence detected in over 50% of Glps, characteristically involved in protein-protein interactions was also present [51]. In animal cells, these tripeptides domain is found in cell adhesion proteins from the extracellular matrix (such as vitronectin and fibronectin) that interact with transmembrane proteins called integrins.

The putative phosphorylation sites in Ma-Glp were predicted to be serine at positions 38, 132 and 191 and threonine at position 71. Serine at position 132 had the major likelihood of phosphorylation as the prediction scores were the greatest compared to the other potential phosphorylation sites [37]. The prediction for N-acetylation was negative as no alanine, glycine, serine or threonine was present at positions 1–3 in the amino acid sequence of Ma-Glp. A putative O-glycosylation site was also predicted at position 70 (threonine) in the sequence. The theoretical pI of the full protein was 6.32 (charge at pH 7.0 = −1.515) and molecular mass of 21,888 Da, whereas the mature protein (without the signal peptide) had a theoretical pI of 6.07 (charge at pH 7.0 = −1.681) and molecular mass of 19,863.7 Da. Apart from the above, the mature protein statistics includes 12 strongly basic (6.28%), 14 strongly acidic (7.33%), 81 hydrophobic (42.41%) and 48 polar (25.13%) amino acid residues[52]. The ORF statistics included %A – 21.27; %G− 22.54; %T− 26.03 and %C− 30.16 with %A+T of 47.3 and %C+G of 52.70 and a melting temperature of 85.71°C. The maximum dinucleotide frequency was for CC at 9.1%, closely followed by CT and TC at 8.9 and 8.7% respectively. Similarly, the maximum trinucleotide frequencies calculated was for CCC and TTT at 3.2%, closely followed by CAA and CTT at 2.9% respectively. The frequent tetramer (9 times) was of CTTT making up of about 1.4% in the ORF (EditSeq, Lasergene). The instability index was computed to be 23.22, which classified the protein to be stable, whereas the aliphatic index was found to be 99 [53]. Also the general average of hydropathicity (GRAVY) plot, calculated as sum of hydropathy values of all amino acid residues in sequence was 0.387 [54]. The extinction coefficient was computed to be 7575 M^−1^cm^−1^ at 280 nm measured in water. This is assuming all pairs of Cys residues form cystines [55]. The most significant residue absent in the deduced amino acid sequence of Ma-Glp was Tryptophan (Trp). Normally proteins without Trp residues can form about 10% error in the extinction coefficient predictions.

A phylogenetic tree, comprising of 28 germin/Glp sequences from 16 species (Table 2), was generated (Fig. 13B). The dendrogram analysis defines that germins/Glps could be divided into major groups of true germins and Glps. It is also appropriate to subdivide the Glps into three main subfamilies (I, II and III). Ma-Glp has been strategically placed in subfamily 3 of Glps that is certainly the largest of the three groups. Though the functional significance of Ma-Glp is yet to be established, studies have extensively reported the existent correlation between phylogenesis and functional properties of these groups and subgroups. The true germin family members comprise of proteins having oxalate oxidase (OXO) enzyme activity catalyzing the manganese-dependent oxidative decarboxylation of oxalate to carbon dioxide and hydrogen peroxide [56], whereas Glp gene members corresponding to subfamilies I and II code for proteins exhibiting superoxide dismutase (SOD) activity [57]–[59]. A homohexameric barley germin, for which 3D structure has been resolved, displays both OXO and SOD activities [47], [60]. The enzyme activity of Ma-Glp needs to be elucidated but its presence in subfamily III may direct it as a regulatory protein involved directly or indirectly in auxin metabolism [61], [62]. This is also significant as the percent identity matrix (Fig. 13C) has revealed its greatest similarity with *Prunus salicina* Glp1 (72%) and *Prunus persica* ABP19a (71%), followed by *Prunus salicina* Glp2 (67%), *Prunus persica* ABP20 and *Vitis vinifera* Glp6 (66%). This group includes low-affinity auxin-binding proteins from peach [63], [64], cotton Glp1 important for cell wall expansion [65], Glps linked to circadian rhythms as in *Arabidopsis* [66], *Hordeum* [67], *Sinapis* [68] and *Pharbitis* [69]. It is important to note here that though there exists strong sequence diversity among the germins/Glp subfamilies; most of them confer resistance against pathogen infection [70], [56].

The existence of the proteolytically processed form of Ma-Glp in silkworm fecal matter as the predominant form of protein and its stability at a higher temperature confirms the thermal tolerance andalkaline resistant attribute of the protein previously demonstrated [71], [72].The most suitable interpretation would be that the midgut-active defensive proteins are highly resistant to digestive proteases, and as a consequence, are selectively enriched during passage of the food bolus through the animal. Also, the stability of proteins in the gut may be due to herbivory induced post-translational modifications, regulating their defensive function [73]. It therefore may have been preserved in the insect physiological system and may form critical structural components of the insect digestive apparatus.

These genes have been found to be expressed in response to attack by fungal pathogens, bacteria and viruses, while expressing in wide range of plant tissues, acting against parasites like nematodes [74]–[76].Reports of the processed form of enzyme threonine deaminase 2 (TD2), activated by the jasmonite signaling pathway in response to herbivore attack in tomato (*Solanum lycopersicum*) have been catalogued from the feces of *Manduca sexta* and *Trichoplusia ni* [77].The report also emphasized the cataloguing of a Glp in the extract, similar to a Glp isozyme from *N. attenuata* that has a role to play in resistance to *M. sexta* attack [78]. This helps us to assume that detection of Glps in feces may help to exert defensive effects (e.g. H_2_O_2_ production) in the herbivore gut. This also makes an attractive subject of research towards understanding the evolutionary origins of plant enzymes that exert toxic or antinutritional effects on insect herbivores.

*Ma-Glp* belongs to the cupin superfamily of proteins, named on the basis of a conserved β-barrel fold (classic jelly-roll beta-barrel structural domain) and was originally discovered using a conserved motif found within germin and Glps [48]. It would belong to monocupins subgroup having a single cupin domain at the centre of the protein, mostly comprising of OXO enzymes in plants, along with proteins such as microbial phosphomannose isomerases and AraC-type transcriptional regulators. Also, cupin superfamily can bind a number of metal ions including manganese, iron, zinc and copper. However, cupin proteins have all been known to only bind to mononuclear metal ions, making it more likely that *Ma-Glp* to be a manganese containing protein.

### Secondary structure prediction of Ma-Glp

**Figure 15:**
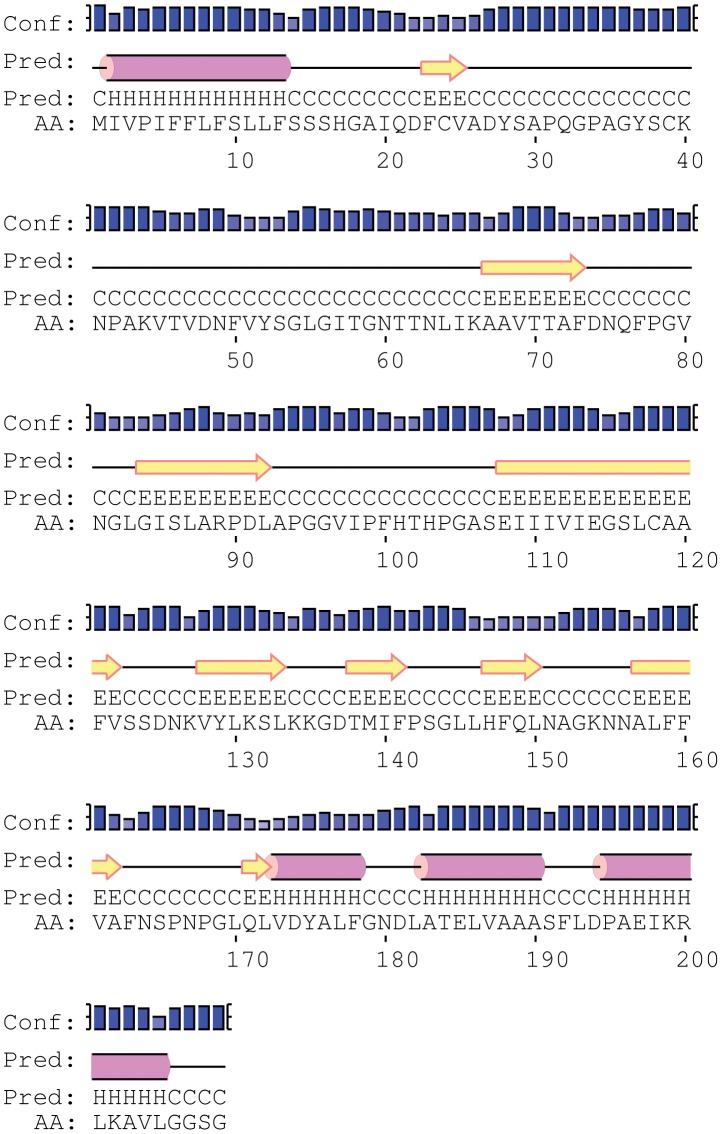
Secondary structure prediction for Ma-Glp. Secondary structure prediction was performed based on position-specific scoring matrices using the PSIPRED method. The sequences marked as ‘H’, ‘E’ and ‘C’ corresponds to helix, extended strand state and random coil state respectively.

The insights to secondary structure prediction of novel Glp from *M. alba*, has predicted α-helices at the signal peptide region and extreme C-terminus of the protein, but have ahigh content of beta pleated sheets (Fig. 15). Most of these conserved β-structures are flanked by absolutely conserved aa residues, most frequently glycine. Conservation of glycine residues is related to the flexibility in enzyme structure and function [79]. The presence of tightly packed hydrophobic residues and potential formation of isoleucine clusters (9 out of 12) in the β-barrel region and number and location of proline residues are regarded to play an important role in increasing the thermal stability of the protein [80]. The number of proline residues in Ma-Glp was found to be 13, i.e. 6.2% of total residues (a value in closer proximity to that of thermophilic proteins), out of which 10 were present in the extended loop region (Fig. 15). The presence of proline residues in the loop regions might have stabilized and made the loops resistant to be cleaved by proteases. The observation is ably supported by site-directed mutagenesis studies with ribonuclease A, where Ala20 substitution by Pro in the loop region, increased the proteolytic resistance of the enzyme [81].

**Figure 16:**
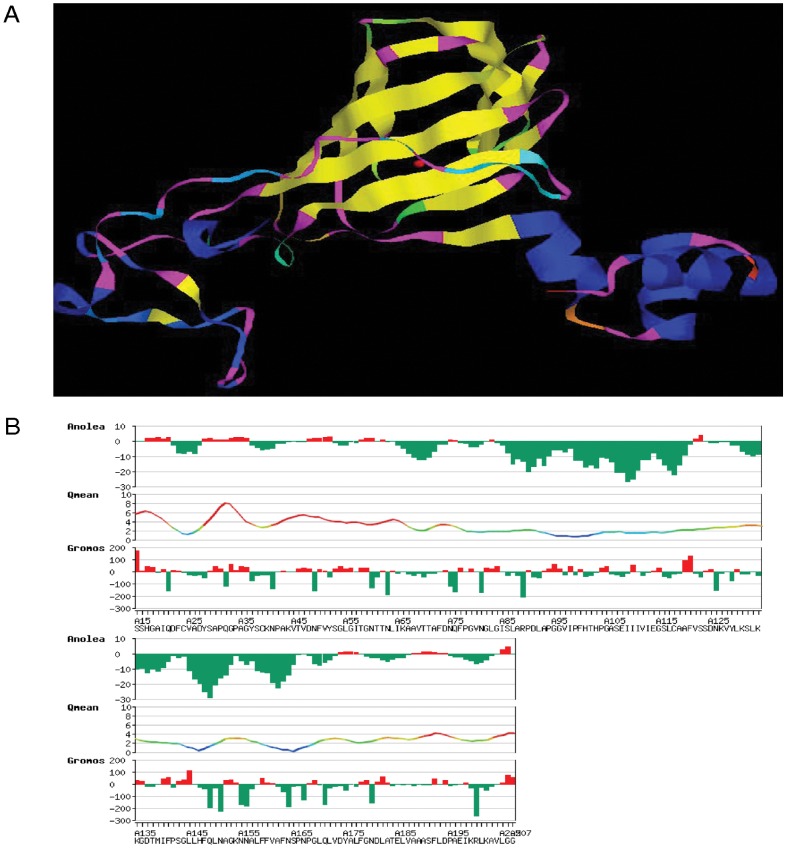
Homology modeling (theoretical model prediction) using SWISS-MODEL workspace at expasy.org. (A) The model was based on the germin (oxalate oxidase) template [1fi2A] at 1.60 A resolution having a QMean Z score of −3.88 and QMean score 4 of 0.5. The modeled residue range was from 15–208. The predicted structure is visualized using RasWin Molecular Graphics Version 2.7.5. The α-helices and β-sheets are represented blue and yellow in color respectively. The red atom at the centre of the model corresponds to the metal manganese. The beta-sheets are arranged in the β-barrel format. (B) The local model quality estimation by ANOLEA (atomic non-local environment assessment) value.

The homology modeling of the Ma-Glp based on the crystallographic evidence data carried out on a barley germin protein revealed a beta-barrel confirmation to the protein which are already evidenced as characteristic to cupin superfamily with hetero atom (Manganese) binding site conserved within the germin boxes B and C (Fig. 16A). ANOLEA was used in the Swiss-Model procedure (Fig. 16B) to assess the quality of the model (Ma-Glp protein). The ANOLEA value of the residues of the Ma-Glp protein was <0, for residues in the N-terminal region. This may relate to the steric hindrances of the residues and may not be perfect. Apart from those, the regions of the mature protein had a lower value. According to protocol, the lower the ANOLEA value, the more accurate the predicted structure of model [82], [83]. From the model estimation data, the predicted structure of the model seemed reasonable.

The crystal structure of barley germin protein also has confirmed that six germin proteins (which each bind a single manganese-ion) make up a extremely stable hexamer protein structure [47]. Each germin protein ‘monomer’ binds to another creating a hexamer structure made of ‘trimer of dimers. Each germin monomer is comprised of an irregular N-terminal extension, the beta-barrel and a C-terminal sequence containing three alpha helices. This is also true to Ma-Glp structure prediction using the most reliable prediction method based on position specific scoring matrices [84]. Interestingly, though the irregular N-terminal domain shape is conserved in Glps. Also, in total the hexamer contains about 1,200 amino acids with an approximate molecular mass of 130 kDa [85].

## Conclusion

The present study reports the characterization of novel Glp from white mulberry, *M. alba* by the method of purification of its mature form from silkworm fecal matter. The N-terminal amino acid sequence of the purified protein extracted from silkworm fecal matter was found exactly similar to the deduced amino acid sequence (without the transit peptide sequence) of the full length cDNA from the food plant *M. alba*. The protein was found stable at extreme temperature and showed activity against some critical bacterial and fungal strains. The activity was maintained at high temperatures that conform to the character of Glps as thermally resistant proteins showing intricate functions in both biotic and abiotic stresses in plant system. It may have evolutionary significance in the insect system as forming structural components of the digestive system. The Ma-Glp had the primary and secondary structure attributes similar to other germins/Glps and was classified under subfamily 3 of Glps. It is important to note further the OXO or SOD enzyme activity of the novel germin to act as a important factor in resistance to pathogenic fungus and other herbivores insects.Excretion of processed active form of Glp from *B. mori*, leads to the hypothesis that insect feces may provide a rich source of material in which to identify other defense-related proteins. It is therefore concluded that, proteomic analysis of fecal extracts would provide with a robust experimental approach towards identification of hyperstable (as well as alkaliphilic) plant proteins that serves important roles in defense as well as to demonstrate the fate of plant proteome interacting with the components of the insect gut. Additionally, the fecal extract accumulating plant proteins can be exploited for commercial interest as industrial biocatalysts.
